# Improving Severe Combined Immunodeficiency and Cystic Fibrosis Newborn Screening by Replacing Current Tests with First-Tier Targeted Gene Sequencing

**DOI:** 10.3390/ijns12030056

**Published:** 2026-07-23

**Authors:** Bennett O. V. Shum, Carel Pretorius, Ilya Henner, Emre Basar, Urs Wilgen, Glenn Bennett, Jacobus P. J. Ungerer

**Affiliations:** 1 Preventive Health Division, Genepath, Sydney, NSW 2067, Australia; 2 EMBL Australia Node in Single Molecule Science, School of Biomedical Sciences, University of NSW, Sydney, NSW 2052, Australia; 3 Department of Chemical Pathology, Pathology Queensland, Queensland Health, Brisbane, QLD 4029, Australia; 4 Faculty of Health and Behavioural Sciences, University of QLD, Brisbane, QLD 4072, Australia

**Keywords:** genomic newborn screening, targeted genomics, targeted gene sequencing, next-generation DNA sequencing, next-generation sequencing, newborn screening, newborn bloodspot screening

## Abstract

Newborn screening benefits children with rare diseases by enabling early detection and intervention, which improves health outcomes. False-positive results are one harm of screening, with infants subject to further testing to resolve a positive screen and parents susceptible to psychosocial distress while awaiting the results. Newborn bloodspot screening (NBS) for severe combined immunodeficiency (SCID) using quantitative polymerase chain reaction (qPCR) and cystic fibrosis (CF) screening using immunoreactive trypsinogen (IRT) have high false-positive rates, with more than five infants screening positive and unaffected, for each child diagnosed. We used first-tier targeted gene sequencing (TGS) to prospectively screen 3025 newborns for CF, SCID and spinal muscular atrophy (SMA) and compared the results to current screening protocols using multiplex qPCR and IRT. TGS identified one infant with CF, missed by the IRT assay, and detected an infant with SMA, who screened positive with multiplex qPCR. TGS had zero false-positive results for SCID, CF and SMA. By contrast, multiplex qPCR and IRT screening protocols had approximately nine false positives for each child diagnosed with CF (positive predictive value, PPV 10.00%) and SCID (PPV 10.13%). Replacing current SCID and CF screening protocols with TGS would reduce NBS false positives and may result in a higher sensitivity for CF. TGS sensitivity and specificity would be comparable to qPCR for SMA. A larger trial is required to determine TGS sensitivity and cost-effectiveness.

## 1. Introduction

Newborn bloodspot screening (NBS) programs across Australia screen more than 99% of approximately 300,000 infants born annually. The programs are guided by a National Policy Framework [1], based on Wilson and Jungner criteria [2], ensuring that conditions screened have an evidence base and national consensus. Currently, 37 conditions are screened nationwide [3], including severe combined immunodeficiency (SCID), spinal muscular atrophy (SMA), and cystic fibrosis (CF). In addition, 18 non-target conditions, which do not meet screening criteria, may be incidentally detected.

Since the introduction of phenylketonuria screening in the 1960s [4], NBS in Australia has evolved substantially. By 1999, all programs screened for congenital hypothyroidism and CF, and most also screened for galactosaemia, each requiring separate, disease-specific assays. A major advancement occurred in 1998 with the introduction of multiplex tandem mass spectrometry, enabling simultaneous, cost-effective detection of multiple metabolic disorders in one test [5]. This technology was implemented nationwide by 2005. More recently, a multiplex quantitative polymerase chain reaction (qPCR) assay for SCID and SMA was introduced, with nation-wide implementation achieved by 2023.

Most conditions included in NBS programs are monogenic with Mendelian inheritance, prompting interest in utilizing next-generation DNA sequencing (NGS) for screening [6]. NGS enables multiple genes to be simultaneously analyzed for disease-causing variants, potentially detecting numerous genetic conditions in a single assay. Multiplex NGS testing may optimize current NBS protocols by replacing multiple disease-specific assays and could be used to screen conditions meeting established criteria but lacking a suitable assay [7].

NGS methods include targeted gene sequencing (TGS), whole-exome sequencing (WES), and whole-genome sequencing (WGS). TGS can be tailored to only analyze genes responsible for conditions that satisfy NBS criteria, minimizing incidental findings. In contrast, WES and WGS analyze the entire exome or genome, over 20,000 genes, however over 99% of the data generated comes from genes unrelated to diseases meeting screening criteria and are excluded from interpretation. Over the past decade, quantitative studies utilizing TGS have outnumbered those employing WES or WGS [8], and demonstrated the advantages of TGS with respect to cost-effectiveness, scalability, privacy and consent [9]. While there is substantial qualitative research on WGS, questions about cost and feasibility persist due to a lack of quantitative data [10].

SCID is one condition that may be amenable to screening using first-tier TGS. It has 20 causative genes which mostly exhibit autosomal recessive inheritance, except for *ILR2* which is X-linked, and *RAC2* which is autosomal dominant [11]. *RAC2* loss-of-function variants can also cause SCID-like disorders, inherited in either an autosomal dominant [12] or autosomal recessive [13] manner, which are exceptionally rare and do not have a robust evidence base supporting screening.

In NBS programs, SCID is typically screened using a qPCR assay that quantifies T-cell receptor excision circles (TRECs), and often multiplexed with SMA detection for cost-effectiveness. While TREC measurement is highly sensitive, it lacks specificity and identifies more infants with non-SCID-related low TREC levels than those actually affected [14]. Many infants with low TREC levels have conditions like DiGeorge syndrome, or other secondary causes, while some have no apparent underlying cause, making decisions regarding intervention a challenge [15]. In many cases, low TREC results are analytical false positives, with normal T-cell counts and no clinical explanation [16]. Although these false positives are generally not associated with long-term adverse outcomes [17], they impose a significant burden on families and healthcare systems. Currently, the high rate of false positives from the TREC assay remains a major barrier to implementing NBS for SCID in the United Kingdom [18].

Reported TREC false-positive rates range from 0.01% to 0.09% [16]. Strategies to reduce false positives include adjusting cut-offs, repeat testing with a second dried bloodspot sample, and modified algorithms for premature infants. Some programs also employ second-tier molecular testing to improve specificity [16,19]. In the Queensland NBS program, initial positive TREC results are followed by repeat sampling, with persistent abnormalities prompting referral for diagnostic flow cytometry.

CF is another condition for which first-tier TGS may offer advantages. CF is caused by pathogenic variants in the *CFTR* gene which are inherited in an autosomal recessive manner [20] and is currently screened using immunoreactive trypsinogen (IRT) as a biomarker. Although IRT is sensitive, it may miss 2.5% to 13.4% of affected infants [21] and lacks specificity, generating high false-positive rates [22].

To address this, most programs incorporate genetic testing for *CFTR* variants in their CF screening protocol. Genetic tests which identify a common CF-causing variant are mostly used, and comprehensive *CFTR* sequencing as a second-tier [23] or third-tier test [24] is infrequently undertaken. In Queensland, infants with two variants identified by a test for 50 common *CFTR* variants are referred for diagnostic sweat chloride testing. Because genetic tests using limited variant panels fail to detect rare variations, infants with one variant are also referred for sweat testing, resulting in a high number of false-positive results from the CF screening protocol due to unaffected carriers. Even with second- or third-tier genetic testing, approximately five to six false positives are identified for each confirmed case [22].

The identification of carrier status through CF screening presents an ethical dilemma for NBS programs [25], because releasing results provides no immediate benefit to the infant but the information is often valued by families [26]. Although false-positive results are not typically associated with long-term harm [17], false negative CF NBS, while uncommon, can result in delayed diagnosis and poorer clinical outcomes [27]. These limitations of the IRT assay have prompted ongoing work to improve CF screening [28].

SMA represents a third condition that is potentially suitable for first-tier TGS. Approximately 95% of affected infants have a homozygous deletion of exon 7 in the *SMN1* gene, which can be reliably detected by targeted sequencing [29]. Current qPCR-based screening demonstrates high sensitivity and specificity, with false positives being rare [30]. However, integrating SMA screening with SCID and CF into a single TGS assay may offer efficiencies over existing multi-platform approaches.

Previous work from our group has demonstrated the feasibility of first-tier TGS for NBS. Analytical sensitivity and specificity exceed 99%, and in a cohort of 2552 newborns, TGS achieved 100% clinical specificity for CF, SCID, and SMA, with no false-positive results [9,31]. TGS also enables comprehensive *CFTR* analysis, including the detection of rare variants not captured by standard DNA screening panels [32], and, with custom bioinformatics, can screen for SMA [29].

The objective of this study was to evaluate the performance of a first-tier TGS approach in a prospective cohort, compared with current IRT-based CF screening and multiplex qPCR-based SCID and SMA screening. Specifically, we aimed to (i) scale a TGS platform for high throughput, real-time detection of single-nucleotide variants, insertions/deletions, and copy number variants, and (ii) assess its clinical sensitivity and specificity.

## 2. Materials and Methods

### 2.1. Study Design, Setting, and Population

This single-site, prospective observational study compared standard NBS protocols for CF, SCID and SMA with a first-tier TGS assay. The study was approved by the Metro North Human Research Ethics Committee, Brisbane, Australia (HREC/2020/QRBW/64125; 18 May 2020).

### 2.2. Multiplex qPCR SCID/SMA Screening

Over an 8-month period, 40,558 newborns underwent SCID and SMA screening using a first-tier multiplex qPCR assay. Consent for testing was obtained from parents and dried bloodspot (DBS) samples were collected at 48–72 h of life following standard Queensland NBS procedures. Testing was performed on the Eonis™ Q96 analyser (Revvity, Waltham, MA, USA).

A positive SCID screen (TRECs < 50 copies/10^5^ cells) prompted repeat DBS collection and qPCR testing. Persistently low TRECs resulted in referral for flow cytometry. A positive SMA screen (homozygous deletion of exon 7 in *SMN1*) was confirmed by multiplex ligation-dependent probe amplification (MLPA; Asuragen, TX, USA).

SCID true positives included infants meeting diagnostic criteria for SCID or T-cell-related lymphocyte deficiencies following flow cytometry, consistent with published guidelines [33]. T-cell-related lymphocyte deficiencies (TCL) were considered a true positive due to clinical follow-up requirements [29]. False positives included infants with normal repeat TREC results or normal flow cytometry. True negatives had normal TRECs and no subsequent diagnosis, while false negatives were those with negative screening but later diagnosis (to December 2025).

SMA true positives were confirmed by MLPA. False positives had no *SMN1* exon 7 deletion on confirmatory testing. True negatives had negative screening and no subsequent diagnosis, and false negatives were identified through later diagnosis.

### 2.3. IRT/DNA CF Screening

Over a 12-month period, 58,062 newborns underwent CF screening using an IRT-based protocol. Parental consent and DBS collection followed standard Queensland NBS procedures. IRT testing was performed on a GSP analyzer (Revvity).

Samples with IRT values above a floating 97.5th percentile underwent second-tier DNA testing for 50 common *CFTR* variants (CFEU2v1; Elucigene, Manchester, UK) using the original DBS sample. Infants with one or two variants were referred for sweat chloride testing.

True positives were defined as a positive sweat test (≥60 mmol/L), CF screen-positive inconclusive diagnosis (CFSPID; 30–59 mmol/L), or two pathogenic or likely pathogenic CF variants. CFSPID cases were included due to clinical follow-up requirements [34]. False positives had normal sweat chloride (<30 mmol/L). True negatives had normal IRT or no variants detected. False negatives were those with negative screening but subsequent CF diagnosis (to December 2025). Samples with incomplete diagnostic follow-up were excluded.

### 2.4. TGS Screening

A subset of 3025 newborns from the SCID, SMA and CF screening population was randomly selected for concurrent TGS testing. Parental consent and DBS collection followed standard Queensland NBS procedures.

A positive TGS result was defined as the presence of pathogenic or likely pathogenic variants in a disease-causing configuration in genes causative of SCID, SMA, or CF.

Case definitions mirrored those used in the standard SCID, SMA and CF screening protocols. SCID diagnosis was confirmed by flow cytometry, SMA by MLPA, and CF by sweat chloride testing. True positives, false positives, true negatives, and false negatives were defined consistently with the comparator protocol.

### 2.5. TGS Panel Design

The TGS panel was developed to screen for 29 primary target conditions included in Australian NBS programs as of June 2023. It was designed to detect >95% of newborns with SCID by including 15 causative genes (*ADA*, *AK2*, *CD247*, *CD3D*, *CD3E*, *CORO1A*, *DCLRE1C*, *IL2RG*, *IL7R*, *JAK3*, *LIG4*, *NHEJ1*, *RAC2*, *RAG1*, and *RAG2*) (Supplemental Table S1). Certain SCID genes (*B2M*, *CD3G*, *PRKDC*, *PTPRC*, and *FOXN1*) were not included in the panel because they were deemed to have low clinical utility for screening within the Queensland population. For example, SCID due to *PRKDC* variants is exceptionally rare and has only been found in 6 people worldwide to date [35].

Additionally, the panel includes three genes associated with SCID-like phenotypes (*PNP*, *RMRP*, and *ZAP70*) [11]. These were included based on our previous work, which demonstrated that these genes have high numbers of carriers within the Queensland population [29].

Target regions included all exons with 15-base pair intronic flanking regions, supplemented by selected intronic variants to optimize capture and identify known non-exonic disease-causing variants.

### 2.6. TGS Laboratory Procedures

Genomic DNA was extracted from three 3 mm DBS punches using the Chemagic 360 (Revvity) on Day 1 of testing. Library preparation was performed using custom SureSelect XTHS2 capture (Agilent, Santa Clara, CA, USA) on a Bravo (Agilent) automated platform on days 2–4, followed by sequencing on a NextSeq 2000 (300 cycles; Illumina, San Diego, CA, USA) on days 5–7. Sample tracking and workflow management were performed using Atlas v0.2.2 (Genepath, Sydney, NSW, Australia).

### 2.7. TGS Bioinformatics and Reporting

Data analysis and reporting occurred on days 7–10 of the testing workflow. Primary sequence data were processed using bioinformatic and reporting software (Atlas v0.2.2, Genepath) which was purpose-built to enable high-throughput testing. For secondary analysis, reads were aligned to GRCh38, and single-nucleotide variants and small insertions/deletions were called using GATK (v4.2.2.0, Broad Institute, Kendall Square, MA, USA). Copy number variants were identified using a proprietary algorithm based on read-depth coverage analysis [35]. A second proprietary algorithm was used for SMA detection [29].

Variants underwent semi-automated tertiary analysis. Atlas software allowed NBS pathologists trained in DNA variant interpretation to independently issue reports without the requirement for a genetic pathologist or clinical geneticist. For variants already present in a Pathology Queensland database, classifications were manually confirmed. For new variants, manual classification was conducted using publicly available population frequency databases, in silico analysis scores, ClinVar classifications, and literature reviews. Classification followed the American College of Medical Genetics and Genomics (ACMG) criteria (pathogenic, likely pathogenic, variant of uncertain significance [VUS], likely benign, and benign) [36], with VUS further stratified as previously described [37].

Quaternary Analysis^TM^ involved interrogation of a batch of up to 1536 samples to identify those with potentially pathogenic variants in a disease-causing configuration. A proprietary algorithm was employed to phase two potentially pathogenic or likely pathogenic variants identified within 300 bp of each other in a single gene. High risk samples were prioritized for reporting according to the age of disease onset.

Screen-positive results were reported when pathogenic or likely pathogenic variants were identified in a disease-causing configuration. For *CFTR*, variants of varying clinical consequence (VVCC) were not reported. For SCID, heterozygous gain of function *RAC2* variants were reported and loss-of-function variants, which do not cause SCID, were omitted from reporting. VUS and carrier status were not reported.

### 2.8. TGS Workflow and Validation

The TGS workflow (batch size 1536; turnaround time 7–10 days; sample failure rate zero, annual capacity ~79,872 samples) has been previously described [9]. Analytical performance was assessed using National Institute of Standards and Technology reference materials using a methods-based approach [31]. Clinical validation included testing of CF samples with known variants; SMA validation has been previously reported [29], while SCID clinical validation remains ongoing.

### 2.9. Carrier Frequency Analysis

Carrier frequencies for SCID and CF were determined by combining the study cohort (*n* = 3025) with a previously published cohort of 2552 de-identified newborns who underwent screening for CF and SCID using TGS [9]. The FASTQ files of the 5577 infants were re-analyzed using the Atlas bioinformatic pipeline. SMA carrier frequency was not assessed due to assay limitations.

A carrier was defined as an individual who had one pathogenic or likely pathogenic variant in a disease with autosomal recessive inheritance. For CF, VVCC and poly-T/TG tract variants were not included in calculating carrier frequency rates.

### 2.10. Statistical Analysis

Sensitivity, specificity, and positive predictive value were calculated using standard definitions:

- Sensitivity = true positives/(true positives + false negatives);
- Specificity = true negatives/(true negatives + false positives);
- Positive predictive value = true positives/(true positives + false positives).

## 3. Results

### 3.1. Multiplex qPCR SCID/SMA Screening Perfornamce

Over an 8-month period, 40,558 newborns underwent multiplex qPCR screening for SCID and SMA. A total of 79 infants (0.19%) had a positive SCID screen (Table 1). One infant was diagnosed with SCID based on flow cytometry and was found to be homozygous for the pathogenic variant c.322C>T (p.Arg108*) in the *RAG1* gene in follow-up testing. Seven others were found to have T-cell-related lymphocyte deficiencies including maternal immunosuppression (*n* = 2), DiGeorge syndrome (*n* = 2), protein losing enteropathy (*n* = 1), Noonan syndrome (*n* = 1), and non-immune hydrops (*n* = 1). The remaining 71 (89.97%) were false positives.

### 3.2. IRT/DNA CF Screening Performance

**Figure 1:**
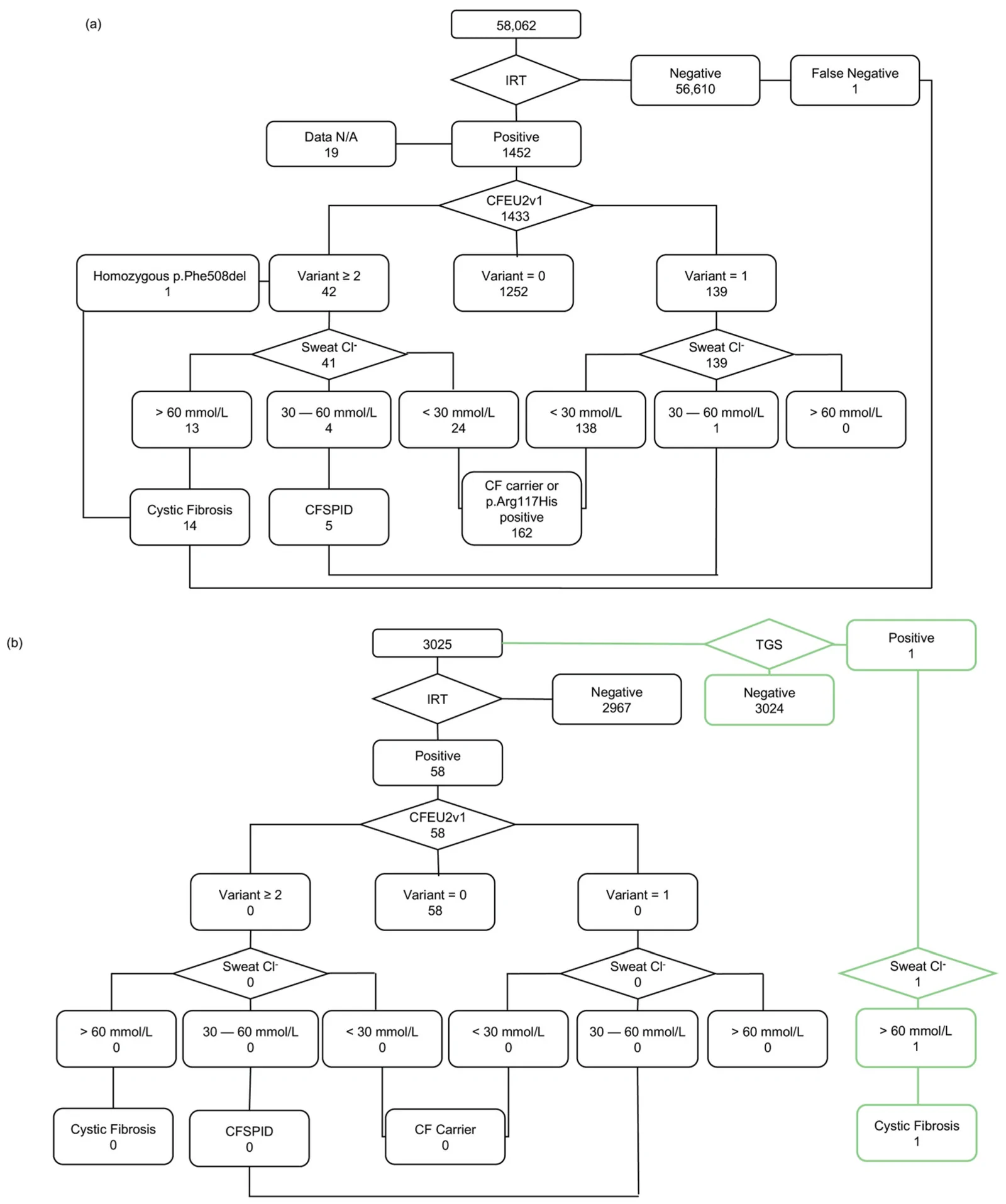
CF screening results. (**a**) Current IRT/DNA screening protocol. DBS samples were screened by an IRT assay. Positive screens underwent second-tier CFEU2v1 testing for 50 CF-causing variants. Patients with one or two variants detected were referred for a diagnostic sweat chloride test. (**b**) TGS screening protocol overlaid with IRT/DNA protocol. A subset of samples from the IRT/DNA screening protocol were screened using first-tier TGS. Only samples with 2 variants were detected by the TGS assay through bioinformatic filtering.

Over a 12-month period, 58,062 newborns underwent CF screening using an IRT-based protocol and 1452 (2.50%) had elevated IRT. Of these, 19 (1.31%) were lost to follow-up and were excluded from analysis, but the remainder (*n* = 1433) proceeded to second-tier DNA testing for 50 common *CFTR* variants. A total of 42 (2.89%) had two or more *CFTR* variants, 139 (9.57%) had one variant, and 1252 (86.23%) had no variants detected (Figure 1). Additionally, 24 of the newborns with two or more variants who had a negative sweat test had VVCC and/or pathogenic poly-T/TG tract variants and were classed as carriers if they harbored a pathogenic or likely pathogenic variant; the remainder were termed p.Arg117His positive.

Of the 42 infants with two or more variants, 24 had normal sweat chloride (<30 mmol/L); all carried at least one low penetrance VVCC or disease-modifying poly-T/TG tract variant. Overall, 180 infants had a positive IRT/DNA screening result, of whom 13 (7.22%) were diagnosed with CF and five (2.78%) with CFSPID. The remaining 162 infants (90.00%) were CF carriers. There was one false negative case (Table 1).

The positive predictive value (PPV) of the IRT/DNA protocol was 10.00% (95% CI 8.44% to 11.81%), with a specificity of 99.72% (95% CI 99.67% to 99.76%).

### 3.3. TGS Screening Performance

A total of 3025 newborns underwent prospective TGS screening. Mean sequencing depth was 263× with 100% coverage uniformity.

One infant screened positive for CF and the diagnosis was confirmed by sweat chloride testing (Figure 1b). This infant harbored two variants, c.3528del (p.Lys1177SerfsTer15) and c.4339del (p.Val1447Ter), both confirmed by Sanger sequencing. One infant screened positive for SMA, confirmed by MLPA. No cases of SCID or T-cell lymphopenia were identified.

There were no false-positive or false-negative results for CF, SCID, or SMA using the TGS protocol.

The PPV for CF and SMA was 100% (95% CI 2.5–100), with a specificity of 100% (95% CI 99.88–100) for both conditions (Table 1). Specificity for SCID was 100%; PPV could not be reliably estimated due to the absence of positive cases.

### 3.4. Concordance Between TGS and Current SCID, SMA, and CF Screening

#### 3.4.1. SCID

Two of the 3025 true negatives by TGS were false positives using multiplex qPCR SCID screening.

#### 3.4.2. SMA

The single true positive and all the true negatives by TGS were correctly identified by multiplex qPCR for SMA.

#### 3.4.3. CF

The single true positive screening result by TGS was not identified using the IRT/DNA CF screening protocol. The IRT of this sample was below cut-off and therefore no DNA testing by the CFEU2v1 DNA test was conducted.

From the 3024 true negatives from the TGS screening protocol, 2966 had a negative IRT and did not have DNA testing via the CFEU2v1 DNA test. The remining 58 samples had a positive IRT and had DNA testing via the CFEU2v1 assay and were reported as having no DNA variants and were not referred for sweat chloride testing. TGS had 100% concordance with the CFEU2v1 results for these 58 samples.

### 3.5. Challenge Samples

Clinical validity was further assessed using five blinded CF positive control samples. TGS correctly identified all variants (5/5, 100%), including a range of variant types: small deletions, missense variants, nonsense variants, splice variants, and copy number variants (Table 2).

### 3.6. Carrier Analysis for SCID and CF

**Figure 2:**
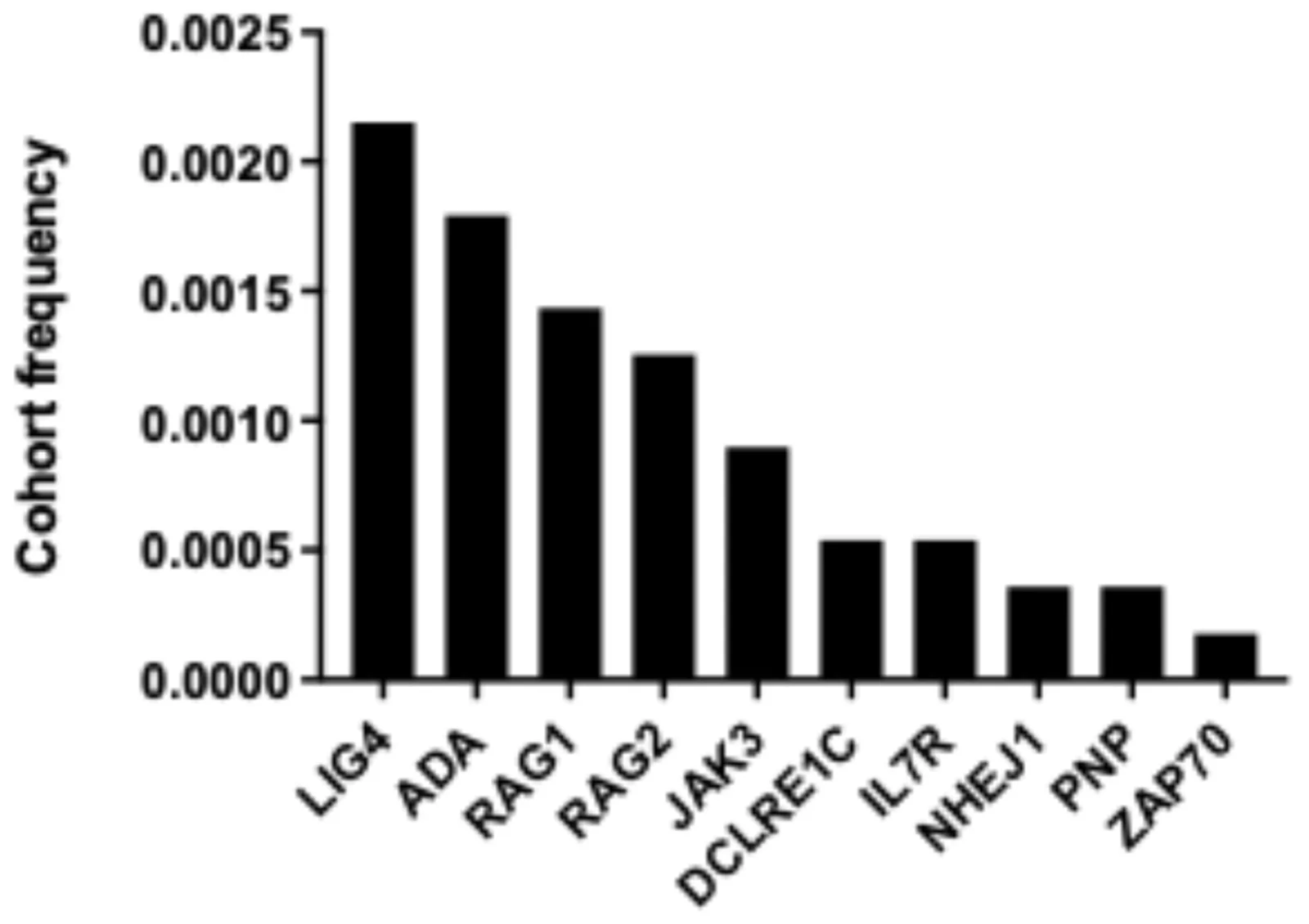
Spectrum of genes causative of SCID and SCID-like syndromes. The number of newborns from our cohort with one likely pathogenic or pathogenic variant in the 15 SCID genes and the 3 genes causative of SCID-like phenotypes are shown. There were no pathogenic or likely pathogenic variants found in *AK2*, *CD247*, *CD3D*, *CD3E*, *CORO1A*, *IL2RG*, *RAC2*, and *RMRP*.

Across a combined cohort of 5577 newborns, approximately one in 105 newborns were carriers of SCID. From the 18 genes causative of SCID and SCID-like phenotypes in the TGS panel, pathogenic or likely pathogenic variants were found for 10 genes (Figure 2).

**Figure 3:**
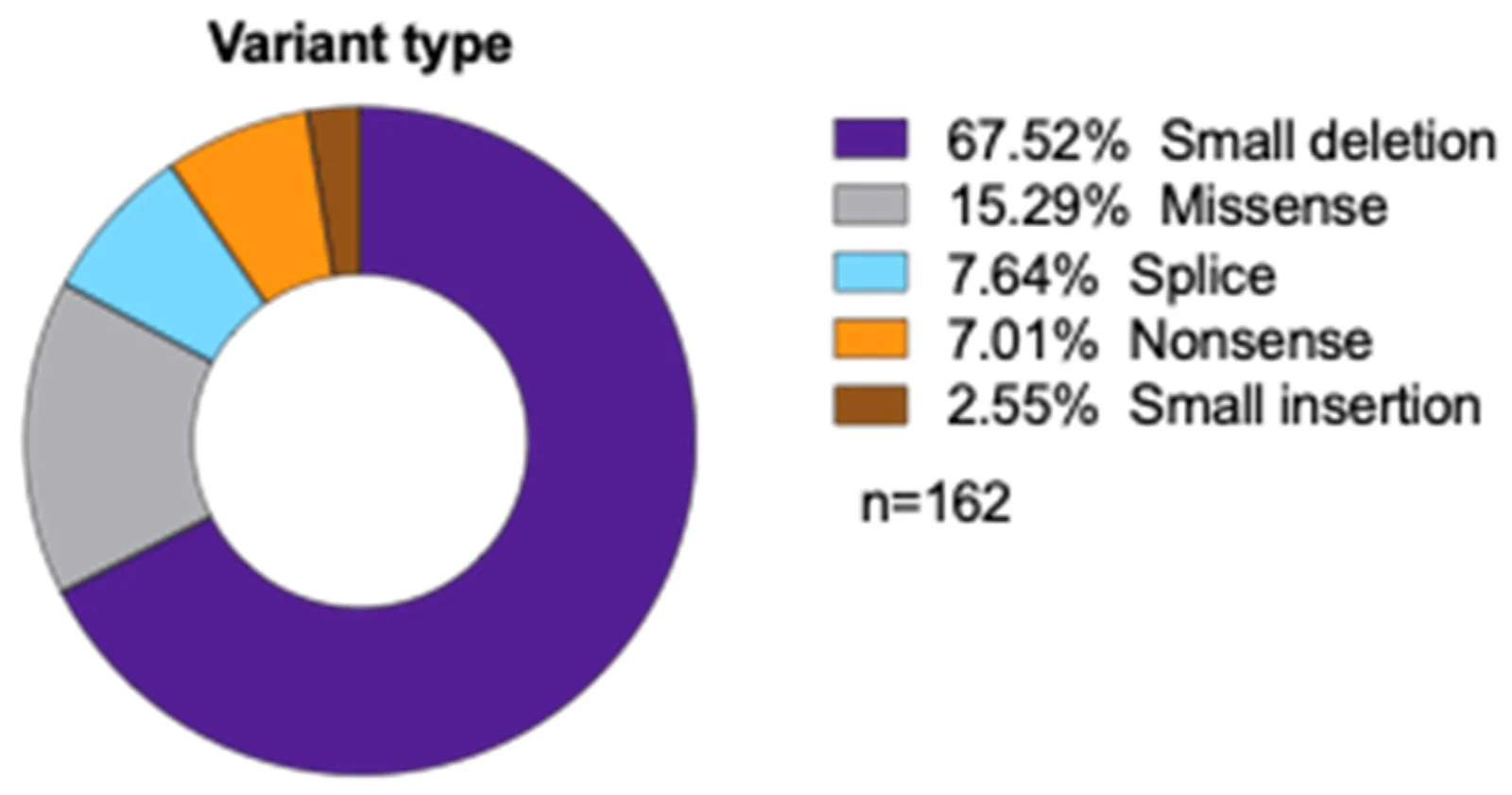
Composition of likely pathogenic and pathogenic *CFTR* variant types. A total of 162 CF-causing alleles found in our cohort were grouped by variant type.

The CF carrier frequency was one in 35. Most CF carriers (67.52%) had small deletions, of which 97.17% were c.1521_1523del (p.Phe508del). Other variant types included missense (15.29%), splice (7.64%), nonsense (7.01%), and small insertions (2.55%) (Figure 3).

A total of 34 CF-causing variants were identified. Notably, 19 variants (55.88%), representing 23 alleles, were not included in the 50-variant CFEU2v1 test (Table 3). Rare variants (allele frequency < 0.0001) collectively represented the second most frequent group, exceeding several common CF variants in the CFEU2v1 test.

## 4. Discussion

We have demonstrated that a TGS assay could be used as a first-tier screening test for a subset of conditions included in the Australian NBS program. Building on our previous work in this area, we have shown that a semi-automated TGS laboratory workflow, supported by a single software platform integrating both wet-laboratory and bioinformatic processes, can achieve high throughput, acceptable turnaround times, and excellent analytical performance for SCID, SMA, and CF. In particular, we demonstrated that first-tier TGS produced no false-positive results for SCID, SMA, or CF, reliably detected SMA, and may offer greater sensitivity than current CF NBS protocols based on IRT. Collectively, these findings suggest that first-tier TGS could replace current IRT-based screening for CF and may also be suitable for SCID and SMA screening.

This study demonstrates that an integrated TGS workflow, supported by software for sample management, data analysis, and reporting, can achieve high-throughput testing within an NBS program. Using a single automated liquid-handling instrument, the system enabled processing of 1536 samples per week with a turnaround time of 7–10 days and a 0% failure rate, resulting in an annual capacity of 79,872 samples. We anticipate that the turnaround time could be reduced to 4–7 days by increasing testing frequency to two batches per week. For programs requiring higher testing volumes, additional automation instruments could be incorporated to support multiple weekly batches and further increase throughput.

We demonstrated that first-tier TGS could improve CF NBS by substantially reducing false-positive results. Although second-tier DNA testing addresses some false positives associated with elevated IRT levels [38], our data indicate that a high proportion of false-positive cases persist under current IRT/DNA screening protocols. In contrast, first-tier TGS eliminated false-positive CF results within our cohort. This is because comprehensive CFTR analysis enabled by TGS ensures that only infants harboring two pathogenic or likely pathogenic variants are referred for sweat chloride testing. Improved specificity has important clinical and psychosocial implications, reducing the need for confirmatory testing and minimizing parental anxiety associated with positive screening results. These benefits may be particularly relevant in geographically large screening programs, such as Queensland, where access to follow-up testing can be logistically challenging.

Importantly, our data indicate that the majority of false-positive IRT results are attributable to analytical and physiological factors rather than carrier status. Only a small proportion of false-positive results generated by the IRT assay alone were associated with CF carrier status. This finding underscores the inherent limitations of biomarker-based screening and supports a transition toward a molecular-first screening approach.

Implementing TGS as a second-tier test following IRT screening would offer greater specificity than many IRT/DNA protocols that do not incorporate comprehensive CFTR analysis; however, it would not provide the potential improvement in sensitivity offered by first-tier TGS. We identified a CF case that was missed by standard screening because of a low IRT concentration but was detected by TGS through identification of a rare CFTR variant. Although this observation is based on a limited sample size, it suggests that TGS may improve sensitivity through its independence from physiological variables. Larger studies are required to determine the magnitude of this effect.

Our analysis of CFTR variation in an Australian cohort further supports the value of comprehensive sequencing within current IRT/DNA screening protocols. Although the common c.1521_1523del variant predominated, rare variants collectively represented the second most frequent group. Many of these variants are not included in CF tests commonly used for second-tier screening, highlighting the limitations of genotyping approaches that focus on common CFTR variants [32]. By capturing the full spectrum of variant types, including single-nucleotide variants, insertions/deletions, and copy number variants, comprehensive CFTR testing has the potential to improve current NBS protocols. This capability may be particularly important in ethnically diverse populations, where rare variants are more prevalent.

In SCID screening, we demonstrated that the high false-positive rates associated with TREC testing can be substantially reduced through implementation of a first-tier TGS approach. Although the TREC assay is highly sensitive, its limited specificity results in many infants without SCID undergoing repeat testing and diagnostic follow-up. Some of these represent analytical false positives, whereas others are diagnosed with TCLs that do not meet accepted NBS criteria. The high false-positive rate associated with TREC testing is a well-recognized limitation [19] and has contributed to the delayed implementation of SCID screening in the United Kingdom [18].

In our cohort, TGS achieved zero false-positive results for SCID. This was possible because the TGS panel was specifically designed to focus on SCID-causing genes while deliberately excluding genes associated with other primary immunodeficiencies. The panel included 15 of the 20 genes classified as SCID-causative by the International Union of Immunological Societies Expert Committee [11], together with three genes associated with SCID-like phenotypes. Although the panel design was not intended to be prescriptive, it illustrates how gene selection directly influences clinical sensitivity and specificity. Theoretically, inclusion of all 20 currently recognized SCID-causing genes in an NBS panel would maximize sensitivity while maintaining a low false-positive rate. Programs wishing to screen for additional primary immunodeficiencies could subsequently elect to include additional genes as required.

Although second-tier genetic testing is known to improve the specificity of TREC-based screening [19], its implementation has been limited by cost. Utilizing TGS as a second-tier test could improve the specificity of current SCID screening protocols; however, multiplexing SCID screening with first-tier genomic screening for CF may offer significant cost-effectiveness advantages. Dedicated health economic studies are required to determine the full magnitude of these benefits.

Our study demonstrates high sensitivity and specificity for SMA screening using TGS. One limitation of the current TGS workflow as a first-tier screening test is the 7–10-day turnaround time, which may delay treatment initiation. Expert consensus recommends that SMA treatment should commence as soon as possible following diagnosis [39]. By increasing testing frequency to two batches per week, we anticipate reducing the turnaround time to approximately 4–7 days. This is comparable to the current qPCR turnaround time of 2–7 days and would support treatment initiation by approximately 20.5 days of age, consistent with the median reported in a recent study [40].

The cost-effectiveness of TGS improves substantially when multiple conditions are screened simultaneously. Consequently, multiplexing SMA with CF and SCID screening may offer considerable advantages. We have previously demonstrated that the cost per diagnosis for TGS can be as low as USD 5262 per positive screen for a 163-gene panel that includes CF, SCID, SMA, and familial hypercholesterolaemia [9]. By comparison, the current cost per diagnosis of SCID alone in Queensland is approximately USD 180,000. The cost-effectiveness of screening for CF, SCID, and SMA using TGS could be further enhanced through inclusion of additional conditions, such as those currently being implemented in the USA (e.g., Duchenne muscular dystrophy) as well as conditions lacking suitable biochemical biomarkers, such as Wilson disease [41].

An important advantage of the TGS approach for NBS is the ability to control the scope of reported findings through purpose-designed bioinformatic filtering. In this study, carrier status was not reported because individuals harboring a single CFTR or SCID-associated gene variant were excluded at the bioinformatic level. This approach avoids the ethical challenges associated with reporting carrier status in newborns [42]. Importantly, the reporting framework is flexible and can be adapted to align with evolving policy requirements and stakeholder preferences.

Although there has been considerable interest in whole-genome sequencing for newborn screening, quantitative data regarding cost, throughput, batch size, and clinical validity remain limited [10]. Our findings provide additional quantitative evidence supporting the role of TGS in NBS. We believe that by focusing on a predefined gene list, TGS avoids many of the costs and the data management, privacy, and ethical challenges that currently prevent whole-genome and whole-exome sequencing from achieving comparable high-throughput performance.

TGS is not without limitations. False-positive CF and SCID results may occur if variants are incorrectly classified as pathogenic. However, this risk can be mitigated through conservative reporting strategies that prioritize well-characterized, highly penetrant variants. Although such an approach may increase the risk of false negatives or reduce detection of cystic fibrosis screen-positive inconclusive diagnosis (CFSPID), integration with clinical assessment and confirmatory testing provides an additional safeguard, similar to existing screening pathways.

Another limitation with assays based on short-read NGS is the inability to comprehensively detect large structural DNA variants such as inversions and translocations which may result in false negatives. However, the vast majority of disease-causing variants in genes covered by our panel are SNVs and small insertions/deletions that are well detected by short reads. Long-read technology offers one solution to detected variants such as inversions; however more research is needed to determine if long-read NGS can be implemented in NBS programs in practical terms and be cost-effective.

One limitation of this study is the lack of a direct cost comparison between TGS and existing screening algorithms for CF, SCID, and SMA, including second-tier testing and downstream clinical follow-up. In a previous publication [9], we estimated the ongoing per-sample cost of a 105-condition TGS panel, including SCID, CF, and SMA, at approximately USD 60, excluding implementation costs. Based on current estimates, this cost has now decreased to approximately USD 40 per sample. By comparison, we estimate that the current per-sample cost of screening for CF, SCID, and SMA using IRT and multiplex qPCR, excluding confirmatory testing, is approximately USD 9.

Our findings suggest that a high-throughput first-tier TGS approach could replace current CF screening protocols and may also be suitable for replacing qPCR-based screening for SCID and SMA within newborn screening programs. A larger trial to determine sensitivity, cost-effectiveness and turnaround time for multi-batch testing is required.

## Figures and Tables

**Table 1:** Performance of multiplex qPCR, IRT/DNA and first-tier TGS screening.

	Multiplex qPCR SCID Screening	Multiplex qPCR SMA Screening	IRT/DNA CF Screening	TGS CF Screening	TGS SMA Screening	TGS SCID Screening
True positive	8	1	18	1	1	0
False positive	71	0	162	0	0	0
False negative	0	0	1	0	0	0
True negative	40,479	40,557	57,862	3024	3024	3025
Sensitivity	100.00% (95% CI 63.06% to 100.00%)	100.00% (95% CI 2.50% to 100.00%)	94.74% (95% CI 73.97% to 99.87%)	100.00% (95% CI 2.50% to 100.00%)	100.00% (95% CI 2.50% to 100.00%)	N/A
Specificity	99.82% (95% CI 99.78% to 99.86%)	100.00% (95% CI 99.99% to 100.00%)	99.72% (95% CI 99.67% to 99.76%)	100.00% (95% CI 99.88% to 100.00%)	100.00% (95% CI 99.88% to 100.00%)	100.00% (95% CI 99.88% to 100.00%)
Positive predictive value	10.13% (95% CI 8.20% to 12.45%)	100.00% (95% CI 99.99% to 100.00%)	10.00% (95% CI 8.44% to 11.81%)	100.00% (95% CI 2.50% to 100.00%)	100.00% (95% CI 2.50% to 100.00%)	N/A

**Table 2:** Performance of TGS with challenge samples.

Sample	Variant One, Type	Variant Two, Type	TGS
1	c.1521_1523delCTT, small deletion	Deletion of exons 2 and 3, CNV	Correct
2	c.1521_1523delCTT, small deletion	c.870-1G>C, SNV	Correct
3	c.1521_1523delCTT, small deletion	c.350G>A, SNV	Correct
4	c.1521_1523delCTT, small deletion	c.1624G>T, SNV	Correct
5	c.1521_1523delCTT, small deletion	c.1652G>A, SNV	Correct

**Table 3:** Spectrum of CF variants from 5577 newborns.

cDNA	Protein	Alleles	Breakdown of CF Carriers (%)	Variant Type	Cohort Frequency	gnomAD ^1^	CFEU2v1 Test for 50 CF-Causing Variants
c.1521_1523del	p.(Phe508del)	103	63.6	Small deletion	0.0340	0.0136	Yes
c.1652G>A	p.(Gly551Asp)	13	8	Missense	0.0043	0.0004	Yes
c.3718-2477C>T	p.?	4	2.5	Splice	0.0013	0.0002	Yes
c.1021_1022dup	p.(Phe342HisfsTer28)	3	1.9	Small insertion	0.0010	0	No
c.1519_1521del	p.(Ile507del)	3	1.9	Small deletion	0.0010	6.81 × 10^−5^	Yes
c.1624G>T	p.(Gly542Ter)	3	1.9	Nonsense	0.0010	0.0003	Yes
c.200C>T	p.(Pro67Leu)	3	1.9	Missense	0.0010	1.17 × 10^−5^	Yes
c.1393-1G>A	p.?	2	1.2	Splice	0.0007	7.30 × 10^−5^	No
c.1585-1G>A	p.?	2	1.2	Splice	0.0007	6.81 × 10^−5^	Yes
c.1657C>T	p.(Arg553Ter)	2	1.2	Nonsense	0.0007	7.91 × 10^−5^	Yes
c.1039C>T	p.(Arg347Cys)	1	0.6	Missense	0.0003	0	No
c.1364C>A	p.(Ala455Glu)	1	0.6	Missense	0.0003	9.12 × 10^−5^	No
c.1364C>T	p.(Ala455Val)	1	0.6	Missense	0.0003	5.38 × 10^−5^	No
c.1418del	p.(Gly473GlufsTer54)	1	0.6	Small deletion	0.0003	0	No
c.148T>C	p.(Ser50Pro)	1	0.6	Missense	0.0003	0	No
c.1753G>T	p.(Glu585Ter)	1	0.6	Nonsense	0.0003	4.89 × 10^−6^	No
c.2053del	p.(Gln685AsnfsTer37)	1	0.6	Small deletion	0.0003	0	No
c.2657+5G>A	p.?	1	0.6	Splice	0.0003	0.0001	Yes
c.2875del	p.(Ala959HisfsTer9)	1	0.6	Small deletion	0.0003	0	No
c.3230T>C	p.(Leu1077Pro)	1	0.6	Missense	0.0003	0	No
c.3472C>T	p.(Arg1158Ter)	1	0.6	Nonsense	0.0003	4.88 × 10^−6^	Yes
c.3484C>T	p.(Arg1162Ter)	1	0.6	Nonsense	0.0003	7.29× 10^−5^	No
c.3528del	p.(Lys1177SerfsTer15)	1	0.6	Small deletion	0.0003	0.0001	Yes
c.3846G>A	p.(Trp1282Ter)	1	0.6	Nonsense	0.0003	6.80 × 10^−5^	Yes
c.3874-4522A>G	p.?	1	0.6	Splice	0.0003	2.85 × 10^−5^	No
c.4300_4301dup	p.(Ser1435GlyfsTer14)	1	0.6	Small insertion	0.0003	0	No
c.4339del	p.(Val1447Ter)	1	0.6	Small deletion	0.0003	0	No
c.4408G>T	p.(Glu1470Ter)	1	0.6	Nonsense	0.0003	0	No
c.489+1G>T	p.?	1	0.6	Splice	0.0003	0.0001	Yes
c.494T>C	p.(Leu165Ser)	1	0.6	Missense	0.0003	0	No
c.579+3A>G	p.?	1	0.6	Splice	0.0003	4.88 × 10^−6^	No
c.617T>G	p.(Leu206Trp)	1	0.6	Missense	0.0003	0.0001	Yes
c.773G>T	p.(Arg258Ile)	1	0.6	Missense	0.000330579	0.0000	No
c.808C>T	p.(Gln270Ter)	1	0.6	Nonsense	0.000330579	0.0000	No

^1^ Allele frequency in gnomAD v4 genome database.

## Data Availability

The raw data supporting the conclusions of this article will be made available by the authors on request.

## Supplementary Materials

The following supporting information can be downloaded at: https://www.mdpi.com/article/10.3390/ijns12030056/s1, Table S1: 15 Causative Genes.
