# Supplementary material for: Improving Severe Combined Immunodeficiency and Cystic Fibrosis Newborn Screening by Replacing Current Tests with First-Tier Targeted Gene Sequencing
Source: Int J Neonatal Screen. 2026 Jul 23;12(3):56. doi: 10.3390/ijns12030056 (PMC13511433; doi:10.3390/ijns12030056)
Supplement: Supplementary file 1 [file IJNS-12-00056-s001.zip › IJNS-4263709-supplementary.pdf]

Supplemental Table S1: 15 Causative Genes.

**Gene**	**Transcript**
ADA	NM_000022.4
AK2	NM_001625.4
CD247	NM_198053.3
CD3D	NM_000732.6
CD3E	NM_000733.4
CFTR	NM_000492.4
CORO1A	NM_007074.4
DCLRE1C	NM_001033855.3
IL2RG	NM_000206.3
IL7R	NM_002185.5
JAK3	NM_000215.4
LIG4	NM_206937.2
NHEJ1	NM_024782.3
PNP	NM_000270.4
RAC2	NM_002872.5
RAG1	NM_000448.3
RAG2	NM_000536.4
RMRP	NR_003051.4
SMN1	NM_000344.4
ZAP70	NM_001079.4
